# Supplementary material for: The Fibrotic Effects of LINC00663 in Human Hepatic Stellate LX-2 Cells and in Bile Duct-Ligated Cholestasis Mice Are Mediated through the Splicing Factor 2-Fibronectin
Source: Cells. 2023 Jan 4;12(2):215. doi: 10.3390/cells12020215 (PMC9857260; doi:10.3390/cells12020215)
Supplement: Supplementary file 1 [file cells-12-00215-s001.zip › cells-2107588-supplementary.pdf]

Supplementary Materials

Supplementary Figures

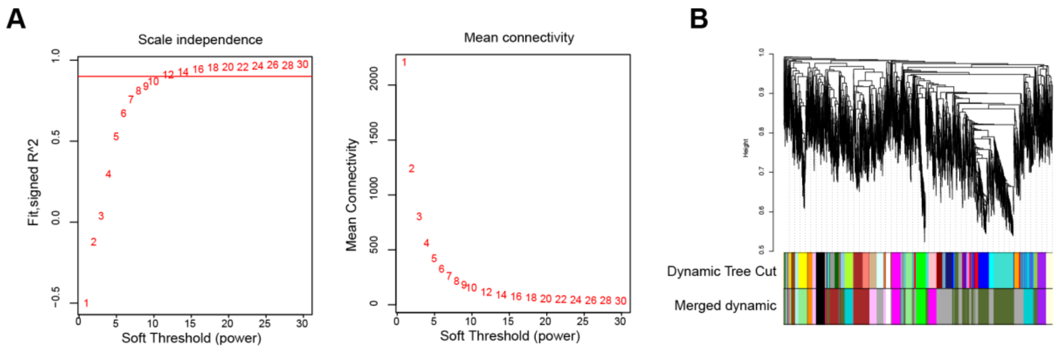

Supplementary Figure S1. Soft threshold filtering and hierarchical clustering diagram. (A) Analysis of network topology for various soft thresholding powers. (B) Clustering dendrogram of genes.

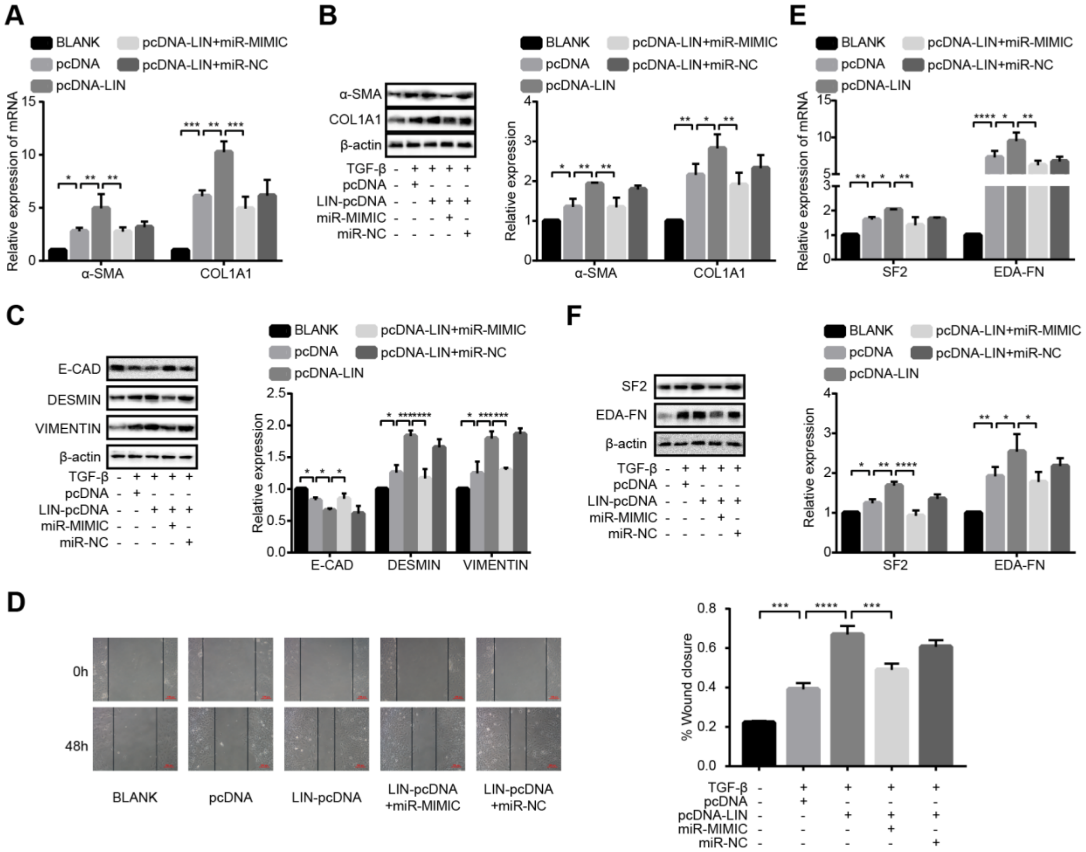

Supplementary Figure S2. LINC00663 regulated activation, EMT, and migration of HSCs through miR-3916. Relative expression of α-SMA and COL1A1 in pcDNA-LIN, miR-mimic or both transfected LX-2 detected by (A) RT–qPCR and (B) WB. (C) Relative expression of EMT protein in pcDNA-LIN, miR-mimic or both transfected LX-2 detected by WB. (D) The effect of LINC00663 and miR-3916 on cell migration in pcDNA-LIN, miR-mimic or both transfected LX-2 detected by wound-healing assay. Scale bar = 100 μm. Relative expression of SF2 and

EDA-FN in pcDNA-LIN, miR-mimic or both transfected LX-2 detected by (E) RT–qPCR and (F) WB. **P*<0.05, ***P*<0.01, ****P*<0.001, *****P*<0.0001; COL1A1, collagen type I alpha 1 chain; EDA-FN, extra domain A of fibronectin; EMT, epithelial–mesenchymal transition; FN, fibronectin; HSCs, hepatic stellate cells; miR, microRNA; SF2, splicing factor 2; α-SMA, α-smooth muscle actin; WB, western blot.

Supplementary Tables

Supplementary Table S1. Sequences of small interfering RNAs of LINC00663, FOXA1, miR-3916 mimic, miR-3916 inhibitor, and their controls.

Name	Sequence（5’- 3’）
SI-NC	sense：UUCUCCGAACGUGUCACGUTT
	antisense：ACGUGACACGUUCGGAGAATT
SI-LINC00663 -1	sense：GCUGGAAUGAGUUAAAUAUTT
	antisense：AUAUUUAACUCAUUCCAGCTT
SI-LINC00663 -2	sense：CAGCGAUGAUGACCGUAAUTT
	antisense：AUUACGGUCAUCAUCGCUGTT
SI-NC	sense：UUCUCCGAACGUGUCACGUTT
	antisense：ACGUGACACGUUCGGAGAATT
SI-FOXA1 -1	sense：GAAGAUGGAAGGGCAUGAATT
	antisense：UUCAUGCCCUUCCAUCUUCTT
SI- FOXA1 -2	sense：GCUCCAUGAACUCCAUGAATT
	antisense：UUCAUGGAGUUCAUGGAGCTT
miR-3916 NC (mimic)	sense：UUCUCCGAACGUGUCACGUTT
	antisense：ACGUGACACGUUCGGAGAATT
miR-3916 mimic	sense：AAGAGGAAGAAAUGGCUGGUUCUCAG
	antisense：GAGAACCAGCCAUUUCUUCCUCUUUU
miR-3916 NC (inhibitor)	CAGUACUUUUGUGUAGUACAA
miR-3916 inhibitor	CUGAGAACCAGCCAUUUCUUCCUCUU

miR, microRNA; SI, small interfering RNAs; NC, negative control; FOXA1, forkhead box A1;

Supplementary Table S2. The connectivity and attribution modules of 116 key genes.

Gene	Eigengene connectivity	Module
Atrnl1	358.9591	darkolivegreen

Gene	Eigengene connectivity	Module
Stmn1	356.0427	darkolivegreen
Fktn	353.9877	darkolivegreen
Bmp2	351.0923	darkolivegreen
C3	350.1919	darkolivegreen
Adra1b	350.1895	darkolivegreen
Cdc6	349.5241	darkolivegreen
Cpe	349.1469	darkolivegreen
Hspb7	347.0505	darkolivegreen
Fzd2	345.77	darkolivegreen
Hist1h2ab	344.5359	darkolivegreen
Tmem119	341.9038	darkolivegreen
Edn1	338.4328	darkolivegreen
Cd200	338.1691	darkolivegreen
Atp1b1	336.8982	darkolivegreen
Gpr39	336.8946	darkolivegreen
Slco2a1	333.8422	darkolivegreen
Dysf	332.6645	darkolivegreen
Top2a	331.8005	darkolivegreen
Cdk1	331.7334	darkolivegreen
Col1a2	331.5381	darkolivegreen
Dpt	331.3855	darkolivegreen
Slc4a4	330.7992	darkolivegreen
Ncam1	330.5481	darkolivegreen
Prkag2	328.565	darkolivegreen
Birc5	328.0173	darkolivegreen
Cadm3	327.0848	darkolivegreen
Parp8	326.3996	darkolivegreen
Srprb	326.1281	darkolivegreen
Lpin2	325.8947	darkolivegreen
Serpinf1	325.5895	darkolivegreen

Gene	Eigengene connectivity	Module
Krt18	325.2842	darkolivegreen
Gins2	323.5097	darkolivegreen
Piezo2	323.1467	darkolivegreen
Mki67	322.6992	darkolivegreen
Prokr1	322.4244	darkolivegreen
Krt8	322.3089	darkolivegreen
Mad2l1	321.9869	darkolivegreen
FN	321.5973	darkolivegreen
Ets1	321.0104	darkolivegreen
Anln	320.7114	darkolivegreen
Abi3bp	320.0143	darkolivegreen
Prc1	319.1624	darkolivegreen
Fcna	318.7486	darkolivegreen
Jph2	317.7922	darkolivegreen
Loxl1	317.301	darkolivegreen
Mfap5	317.2293	darkolivegreen
Lyz1	316.1923	darkolivegreen
Plscr2	315.9047	darkolivegreen
Nuak1	315.7388	darkolivegreen
Fam114a1	315.6929	darkolivegreen
Gpr176	314.9998	darkolivegreen
Creld2	314.4999	darkolivegreen
Alb	314.4648	darkolivegreen
Rab15	314.3905	darkolivegreen
Etnk1	314.2761	darkolivegreen
Actg2	314.271	darkolivegreen
Adora2b	313.785	darkolivegreen
Ugt1a1	313.6336	darkolivegreen
Arhgap6	313.604	darkolivegreen
Plxdc2	312.9817	darkolivegreen

Gene	Eigengene connectivity	Module
Ecm1	312.6654	darkolivegreen
Fbln2	312.3622	darkolivegreen
Ivns1abp	224.9936	darkgrey
Aard	218.3347	darkgrey
Gdf2	216.0943	darkgrey
Plscr4	214.1852	darkgrey
Ch25h	213.1788	darkgrey
AW112010	210.885	darkgrey
Ctso	210.6539	darkgrey
Scp2	207.5769	darkgrey
Gp1bb	206.9672	darkgrey
Vgll3	203.3748	darkgrey
Nr1d2	202.8093	darkgrey
Mmp10	202.6814	darkgrey
AA987161	201.9102	darkgrey
Cotl1	201.9017	darkgrey
Mamdc2	200.571	darkgrey
Ccnyl1	200.003	darkgrey
Arhgap42	199.5246	darkgrey
Reln	199.3077	darkgrey
Fgfr2	198.942	darkgrey
Cdk14	198.7904	darkgrey
Cers6	198.53	darkgrey
Mkrn1	198.4165	darkgrey
Vipr1	198.0945	darkgrey
Nfia	196.8716	darkgrey
Sft2d2	195.7119	darkgrey
Fgf12	194.2663	darkgrey
Camk1	193.478	darkgrey
Pycard	192.5918	darkgrey

Gene	Eigengene connectivity	Module
Ubfd1	192.4515	darkgrey
Nr3c1	191.5825	darkgrey
Ltbp4	191.2194	darkgrey
Lcp1	190.8847	darkgrey
Selenbp1	190.2178	darkgrey
Glis2	189.7655	darkgrey
Reck	189.7577	darkgrey
Tnc	189.7146	darkgrey
Klc3	186.7458	darkgrey
Dtna	186.4076	darkgrey
Mmp13	185.9522	darkgrey
Depdc1a	185.884	darkgrey
Mical2	185.697	darkgrey
Plxnc1	183.2191	darkgrey
Gadd45b	183.1865	darkgrey
Il34	183.0813	darkgrey
Hnmt	183.0323	darkgrey
Tpd52	183.0136	darkgrey
Zfp930	182.527	darkgrey
Ttc19	182.3303	darkgrey
Dtx2	182.2496	darkgrey
Lgals8	182.0762	darkgrey
Tgfbi	181.9422	darkgrey
Gm21949	179.2304	darkgrey
Hspb1	179.1474	darkgrey

Supplementary Table S3. GO analysis of key genes in the co-expression network.

Category	Term	Count	*P*
GOTERM_CC_DIRECT	GO:0005578~proteinaceous extracellular matrix	15	2.98E-09
GOTERM_CC_DIRECT	GO:0031012~extracellular matrix	14	1.16E-08

Category	Term	Count	*P*
GOTERM_CC_DIRECT	GO:0005615~extracellular space	27	2.04E-07
GOTERM_CC_DIRECT	GO:0005576~extracellular region	27	3.71E-06
GOTERM_CC_DIRECT	GO:0070062~extracellular exosome	34	7.38E-06
GOTERM_CC_DIRECT	GO:0005604~basement membrane	6	2.23E-04
GOTERM_CC_DIRECT	GO:0005614~interstitial matrix	3	0.004064
GOTERM_BP_DIRECT	GO:0007155~cell adhesion	10	0.002498
GOTERM_BP_DIRECT	GO:0031100~organ regeneration	4	0.004565
GOTERM_BP_DIRECT	GO:0045766~positive regulation of angiogenesis	5	0.005989
GOTERM_BP_DIRECT	GO:0060045~positive regulation of cardiac muscle cell proliferation	3	0.00824
GOTERM_BP_DIRECT	GO:0033209~tumor necrosis factor-mediated signaling pathway	3	0.009695
GOTERM_MF_DIRECT	GO:0005515~protein binding	47	8.06E-06
GOTERM_MF_DIRECT	GO:0042802~identical protein binding	12	0.001695
GOTERM_MF_DIRECT	GO:0019899~enzyme binding	9	0.002663
GOTERM_MF_DIRECT	GO:0042803~protein homodimerization activity	13	0.003843
GOTERM_MF_DIRECT	GO:0050839~cell adhesion molecule binding	4	0.009721

GO, Gene Ontology

Supplementary Table S4. KEGG analysis of key genes in the co-expression network

Category	Term	Count	*P*
KEGG_PATHWAY	mmu04512:ECM-receptor interaction	5	0.002121
KEGG_PATHWAY	mmu04110:Cell cycle	4	0.041834

KEGG, Kyoto Encyclopedia of Genes and Genomes
